# Prognostic significance of ALCAM (CD166/MEMD) expression in cutaneous melanoma patients

**DOI:** 10.1186/s13000-015-0331-z

**Published:** 2015-07-02

**Authors:** Piotr Donizy, Marcin Zietek, Agnieszka Halon, Marek Leskiewicz, Cyprian Kozyra, Rafal Matkowski

**Affiliations:** Department of Pathomorphology and Oncological Cytology, Wroclaw Medical University, Borowska 213, 50-556 Wroclaw, Poland; Lower Silesian Oncology Centre, pl. Hirszfelda 12, 53-413 Wroclaw, Poland; Department of Statistics, Wroclaw University of Economics, Komandorska 118-120, 53-345 Wroclaw, Poland; Department of Oncology and Division of Surgical Oncology, Wroclaw Medical University, pl. Hirszfelda 12, 53-413 Wroclaw, Poland

**Keywords:** ALCAM, Prognosis, Melanoma, Immunohistochemistry

## Abstract

**Background:**

ALCAM (*activated leukocyte cell adhesion molecule*, CD166, MEMD) is a transmembrane protein of immunoglobulin superfamily (Ig-SF) and plays an important role in human malignant melanoma progression and formation of locoregional and distant metastases. The study using melanoma cell lines showed that overexpression of ALCAM is directly related with the increase of cytoaggregation and the ability to form cell nests. The aim of the study was to assess the expression and intracellular localization of ALCAM in primary skin melanomas and metastatic lesions from regional lymph nodes. Also, prognostic significance of ALCAM expression in primary tumor cells and metastatic lesion cells was evaluated in the context of 5-year observation.

**Methods:**

Formalin-fixed paraffin-embedded tissue specimens from 104 primary cutaneous melanomas and 16 regional lymph nodes metastases were studied for the expression of ALCAM measured by immunohistochemistry.

**Results:**

We demonstrate that high ALCAM expression in primary melanoma cells (IRS ≥8) is strongly correlated with unfavorable prognosis as compared with patients with lower ALCAM immunoreactivity in tumor compartment as regards cancer specific overall survival (CSOS) (*P* = 0.001) and disease free survival (DFS) (*P* < 0.001). Additionally lower ALCAM immunoreactivity in nodal metastatic foci was significantly statistically correlated with deeper melanoma invasion in the primary tumor according to Clark scale (*P* = 0.032). It was also found that decreased ALCAM expression (IRS <8) in nodal metastases shows a trend related with a correlation with shorter cancer specific overall survival (*P* = 0.083). Statistically significant correlations were also demonstrated between the presence of ulceration and decreased intensity of lymphocytic inflammatory infiltration and a high percentage of ALCAM-positive cells (*P* = 0.035, *P* = 0.01, respectively).

**Conclusions:**

High ALCAM expression in melanoma cells of the primary tumor can be used as a marker of negative outcome and may indicate a more invasive phenotype of cancer cells, which would require a more intensive therapeutic strategy. Low expression of ALCAM in regional lymph node metastases is a feature associated with unfavorable prognosis in patients with cutaneous melanoma. Our study is the first one to evaluate the effect of increased ALCAM expression on long-term survival in melanoma patients.

## Background

The unique local homeostasis maintained between keratinocytes, melanocytes and the extracellular matrix that surrounds them has a key role in the regulation of melanocyte growth and proliferation [1, 2]. Each melanocyte is estimated to directly interact and regulate approximately 36 keratinocytes [3]. Melanocyte growth is regulated by the surrounding keratinocytes through various control mechanisms. The two most important involve: (1) paracrine growth factors secreted into the extracellular environment, and (2) intercellular communication of melanocytes and keratinocytes with extracellular matrix proteins via multiple adhesion molecules [4]. All disturbances of this extremely precise homeostasis may lead to abnormal expression of adhesion proteins and molecules involved in intercellular communication, which may in turn initiate melanomagenesis.

In the initial stages of melanoma growth and progression, keratinocytes lose their ability to regulate melanocytes as a result of the three main pathobiochemical processes that occur in melanoma cells. One of them involves reduced expression of receptors such as E-cadherin, P-cadherin and connexin which are crucial for melanocyte-keratinocyte communication [4, 5]. The second mechanism via which the epithelium cells lose control of melanocytes is directly connected with overexpression of receptor proteins and signaling molecules involved in cytological interactions of the melanocyte-fibroblast type. It was demonstrated that increased immunoreactivity of proteins e.g. N-cadherin, Mel-CAM and ZO-1 (*zonula occludens protein-1*) is related with increased invasive potential of melanoma cells [4, 6]. The last mechanism that allows melanocytes to escape from control by keratinocytes involves loss of expression of proteins anchoring melanocytes to the basement membrane [4, 7].

Maintaining intercellular adhesion is a necessary process during invasion of cancer cell nests [8]. However, adhesion deregulation must be one of the stages of metastatic foci development. It is a key moment in which single cells or nests use the changed profile of adhesion molecules expression to occupy new areas, which initiates the spread of cancer. Adhesion protein spectrum whose immunoreactivity panel would be characteristic for invasive melanoma cells has not been identified so far.

ALCAM (*activated leukocyte cell adhesion molecule*, CD166, MEMD) is a transmembrane protein of immunoglobulin superfamily (Ig-SF) [9]. The gene encoding ALCAM is located on the long arm of chromosome 3 and it is organized into 16 exons [10]. Ultrastructural studies show it has 27 % homology with adhesion molecule found on the surface of MUC18/Mel-CAM/CD146 melanoma cells, which promotes progression of this cancer and formation of locoregional and distant metastases [11]. The key element of ALCAM molecule that regulates its adhesive capacity is the extracellular part composed of five immunoglobulin-like domains (D1-D5) [12]. There is also a transmembrane domain and an intracellular short C-terminal tail. At first, ALCAM was identified as a ligand for CD6 receptor (heterotypic interaction, ALCAM-CD6), and its expression was observed on the surface of leukocytes, fibroblasts, epithelial and nerve cells [10, 13]. Other cytophysiological analyses revealed the existence of functional homotypic interactions of the ALCAM-ALCAM type which were identified in CD6-negative melanoma cell lines [14]. The study using melanoma cell lines showed that overexpression of ALCAM is directly related with the increase of cytoaggregation and the ability to form cell nests [14]. Additionally, it was found that the homotypic ALCAM-ALCAM interaction plays a key role during intercellular adhesion. Interestingly, Degen et al. [14] proved that ALCAM expression is correlated with increased metastatic potential of melanoma cell lines.

So far, little is known about regulating expression of ALCAM on the gene level. It was showed that the ALCAM promoter contains a sequence binding NF-κB and AP-1 (*activator protein 1*) which act as transcription factors [15, 16]. Studies into the potential effects of NF-κB on ALCAM expression demonstrated that its binding to the promoter sequence of ALCAM gene enhances the immunoreactivity of the resulting protein product [15]. In turn, the effect of AP-1 family, and specifically overexpression of Fra-2 (*Fos-related antigen 2*) is closely related with the resulting decreased production of ALCAM [16].

Interesting conclusions concerning new biological functions and role of melanoma progression were drawn by Luntner et al. [17] based on their experiment. ALCAM is instrumental for the formation of a triple complex on the surface of melanoma cells MT1-MMP/TIMP-2/pro-MMP-2 and the subsequent conversion of inactive form of metalloproteinase 2 (pro-MMP-2) into an active form responsible for the degradation of extracellular matrix and contributes to the formation of nodal and distant metastases [17]. Inhibition of ALCAM expression resulted in significantly decreased MMP-2 activity, and thus in decreased metastatic potential. Pooled analysis of results has allowed establishing a hypothesis that ALCAM is involved in regulating proteolysis via some still unknown mechanisms and seems to act as a cell-surface sensor for invasive growth [15, 17].

The aim of the study was to assess the expression and intracellular localization of ALCAM in 104 primary skin melanomas and 16 metastatic lesions from regional lymph nodes. Also, prognostic significance of ALCAM expression in primary tumor cells and metastatic lesion cells was evaluated in the context of 5-year observation. Correlations were also analyzed between ALCAM immunoreactivity parameters and detailed clinicopathological parameters such as Breslow thickness and Clark level, presence of nodal and distant metastases, recurrence of primary tumor, mitotic rate, ulceration, lymphocytic inflammatory infiltration, regression and microsatellitosis.

## Methods

### Patients and ethics statements

The study group consisted of 104 patients with CMM, who were diagnosed between 2005 and 2010 and treated in the Lower Silesian Oncology Center in Wrocław, Poland. Additionally, tissue material obtained from 16 nodal metastatic foci was included in the study. The group was selected on the basis of tissue material (paraffin blocks and histopathology slides) and the availability of medical documentation. Comprehensive clinical data were obtained from archival medical records. The diagnostic and therapeutic procedures utilized were determined from medical records in the Oncology Outpatient Clinic of the Lower Silesian Oncology Center and data provided by the Lower Silesian Cancer Registry and Civil Register Office. The retrospective study was approved by the Ethical Committee of the Wroclaw Medical University, Poland.

Patients enrolled in the study were treated by then-available methods. Excisional biopsy of the primary lesion was performed. Once cutaneous melanoma was diagnosed in histopathological examination, the primary procedure was extended by excising the scar with a margin of 5, 10 or 20 mm of unaffected skin depending on Breslow thickness and primary tumor location, if any. If nodal metastases (cN0) were not clinically manifested and Breslow thickness was above 1 mm (>pT1a), sentinel lymph node biopsy (SNLB) was performed. When metastases were observed in the regional lymph nodes (found either clinically or by SLNB), lymphadenectomy was performed.

The clinicopathological profile of patients included the following parameters: age and gender, primary tumor location, tumor stratification according to AJCC (pT), presence or absence of nodal (pN) and distant (pM) metastases, information on disease recurrence and sentinel lymph node biopsy (SLNB) procedures (Table 1).

**Table 1:** Clinicopathological characteristics of the patients

Clinicopathological characteristics	No (%)
All patients	104 (100.0)
Age in years (21–79)	
mean: 56.5 ± 15.4; median: 58.5	
Gender	
Female	60 (57.7)
Male	44 (42.3)
Primary tumor location	
Head/neck	15 (14.4)
Upper extremity	18 (17.3)
Lower extremity	25 (24.0)
Trunk	42 (40.4)
Hand/foot	4 (3.8)
Primary tumor (pT)	
pT1	34 (32.7)
pT2	20 (19.2)
pT3	27 (26.0)
pT4	23 (22.1)
Sentinel lymph node biopsy status (SNLB)	60 (57.7)
No metastases (SNLB-)	48 (80.0)
Metastases present (SNLB+)	12 (20.0)
Regional lymph nodes status (pN)	
No metastases (pN-)	86 (82.7)
Metastases present (pN+)	18 (17.3)
Recurrence	
No	87 (83.7)
Yes	17 (16.3)
Distant metastases	
No	99 (95.2)
Yes	5 (4.8)

### Tumor samples and histopathological evaluation

Tumor specimens were fixed in 10 % buffered formalin and embedded in paraffin. All haematoxylin and eosin stained sections were examined by two pathologists. The parameters of the primary tumor recorded in pathology reports were Breslow thickness, Clark level, growth phase, histologic type, mitotic rate (number of mitotic figures per 1 mm^2^), presence of ulceration, lymphangioinvasion, microsatellitosis, intensity of lymphocytic inflammatory infiltrate (TILs, tumor-infiltrating lymphocytes) and microscopic evidence of regression (Table 2).

**Table 2:** Histopathological parameters of primary tumors

Histopathological parameters of the primary tumor	No (%)
Breslow thickness	
<1 mm	34 (32.7)
1.01-2.00 mm	20 (19.2)
2.01-4.00 mm	27 (26.0)
>4 mm	23 (22.1)
Clark level	
I	0 (0.0)
II	18 (17.3)
III	49 (47.1)
IV	26 (25.0)
V	11 (10.6)
Histologic type	
Superficial spreading melanoma (SSM)	68 (65.4)
Nodular malignant melanoma (NMM)	32 (30.8)
Acral-lentiginous melanoma (ALM)	4 (3.8)
Mitotic rate	
0	45 (43.3)
1-2	26 (25.0)
≥3	33 (31.7)
Ulceration	
No	55 (52.9)
Yes	49 (47.1)
Lymphangioinvasion	
No	74 (71.2)
Yes	30 (28.8)
Growth phase	
Radial	3 (2.9)
Vertical	101 (97.1)
Tumor-infiltrating lymphocytes (TILs)	
No	18 (17.3)
Nonbrisk	34 (32.7)
Brisk	52 (50)
Microsatellitosis	
No	98 (94.2)
Yes	6 (5.8)
Tumor regression	
No	96 (92.3)
Yes	8 (7.7)

### Immunohistochemistry

Formalin-fixed, paraffin embedded tissue was freshly cut (4 μm). The sections were mounted on superfrost slides (Menzel Glaser, Germany), dewaxed with xylene, and gradually hydrated. The activity of endogenous peroxidase was blocked by 5 mins exposure to 3 % H_2_O_2_. The sections were boiled for 15 mins at 250 W in Antigen Retrieval Solution (DakoCytomation, Denmark). Immunohistochemical reactions were then performed using monoclonal antibody detecting ALCAM (clone 10F1G12; Abgent, United Kingdom). The tested sections were incubated with antibodies for 1 h at room temperature. The subsequent incubations involved biotinylated antibodies (15 mins at room temperature) and streptavidin-biotinylated peroxidase complex (15 mins at room temperature) (LSAB+, HRP, DakoCytomation, Denmark). DAB (Vector Laboratories, UK) was used as a chromogen (10 mins at room temperature). All sections were counterstained with Meyer’s hematoxylin. In every case control reactions were performed, with the relevant antibody substituted by Primary Mouse Negative Control (DakoCytomation, Denmark).

### Evaluation of reaction intensity

The intensity of the immunohistochemical reaction was estimated independently by two pathologists. Doubtful cases were re-evaluated under a double-headed microscope and staining was discussed until consensus was achieved.

**Fig. 1:**
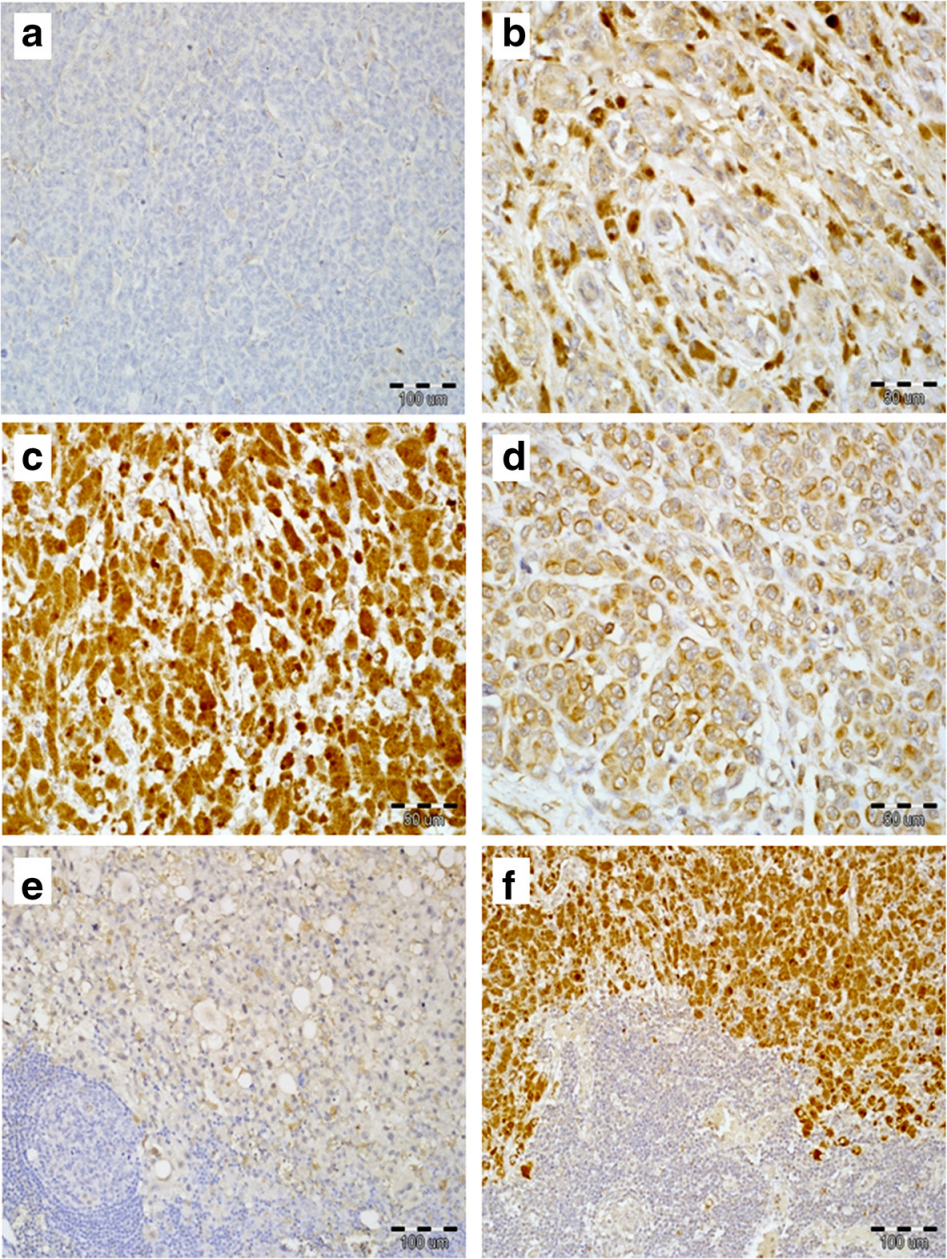
Immunohistochemically visualized expression of ALCAM in cutaneous melanoma. Lack of ALCAM immunoreactivity in melanoma cells from primary tumor (**a**, 200×, hematoxylin). Cytoplasmic distribution of ALCAM in melanoma cells from primary tumor (**b**, IRS 6, 400×, hematoxylin). High cytoplasmic ALCAM reactivity in primary melanoma (**c**, IRS 12, 400×, hematoxylin). Predominantly membranous-cytoplasmic expression of ALCAM in different sizes of nests composed of malignant melanoma cells from primary tumors (**d**, 400×, hematoxylin). Immunohistochemical pattern of ALCAM expression in regional lymph nodes metastases. Low ALCAM immunoreactivity in metastatic melanoma cells (**e**, 400×, hematoxylin). High cytoplasmic reactivity of ALCAM in regional lymph node metastases (**f**, 200×, hematoxylin)

ALCAM expression was observed only in melanoma cells both in tissue material obtained from the primary tumor and nodal metastatic foci. No ALCAM immunoreactivity was identified in stromal compartment of tumor or lymphocytes from regional lymph nodes. Cancer cells of the primary tumor were found to exhibit two patterns of expression. Membranous-cytoplasmic localization was particularly visible in cell nests/cytoaggregates, while diffuse cytoplasmic expression without the membrane component was observed in those cancer cells which did not form evident cell nests but were a pool of scattered melanoma cells (Fig. 1 a-d). Metastatic cells displayed predominantly diffuse cytoplasmic expression (Fig. 1 e-f).

**Fig. 2:**
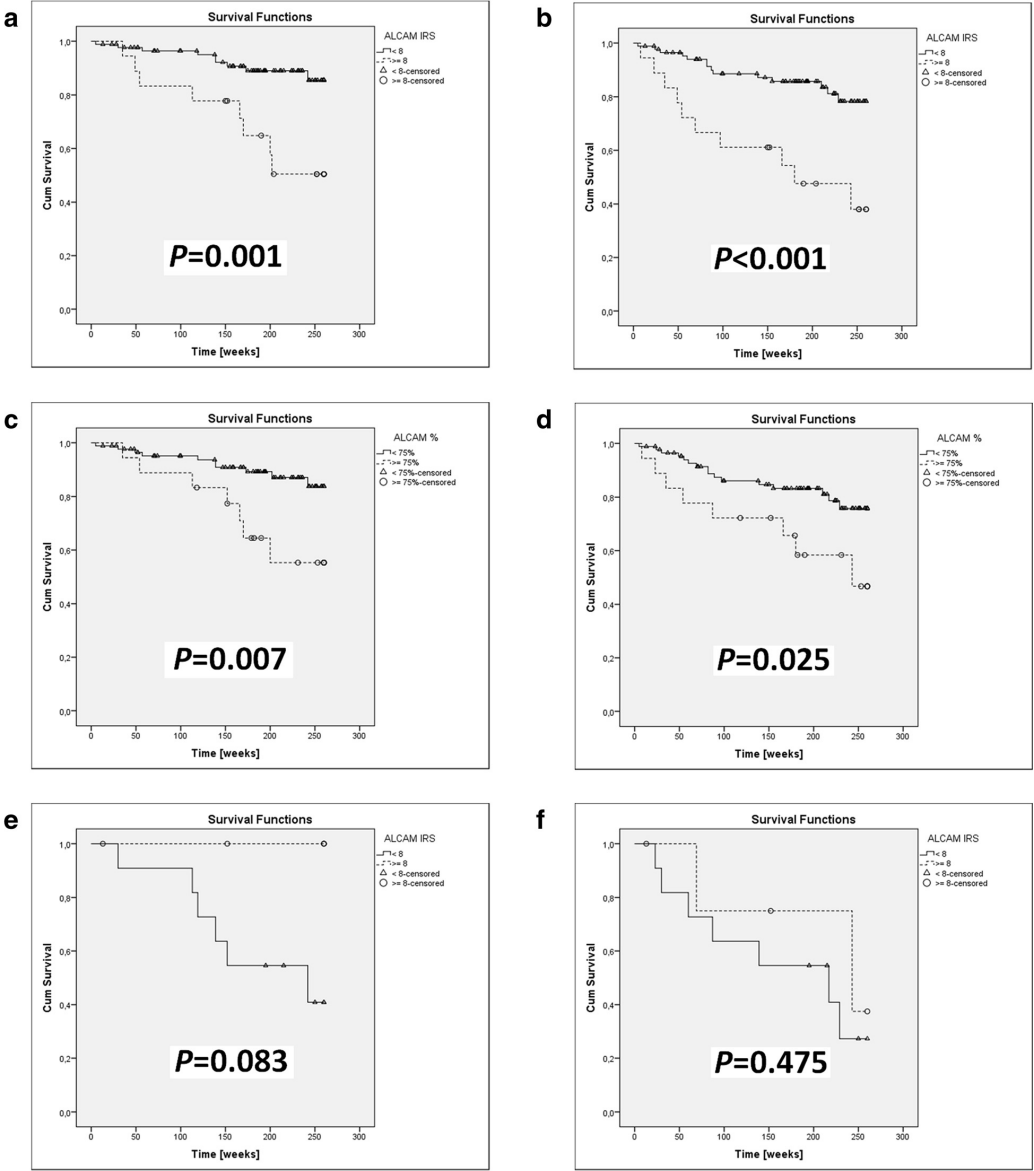
Kaplan-Meier analysis of the impact of ALCAM expression in 104 primary tumors (**a-d**) and 16 nodal metastases (**e, f**) on 5-year survival in melanoma patients. Increased ALCAM expression (IRS) in the primary tumor strongly correlated with shorter CSOS (**a**) and DFS (**b**); analogous associations with shorter CSOS (**c**) and DFS (**d**) were observed for increased percentage of ALCAM-(+) cells in the primary tumor. Survival analysis of 16 cases of nodal metastases revealed a paradoxical trend relating decreased ALCAM expression (IRS) in melanoma metastases with shorter CSOS (**e**), however, there was no association with DFS (**f**)

**Fig. 3:**
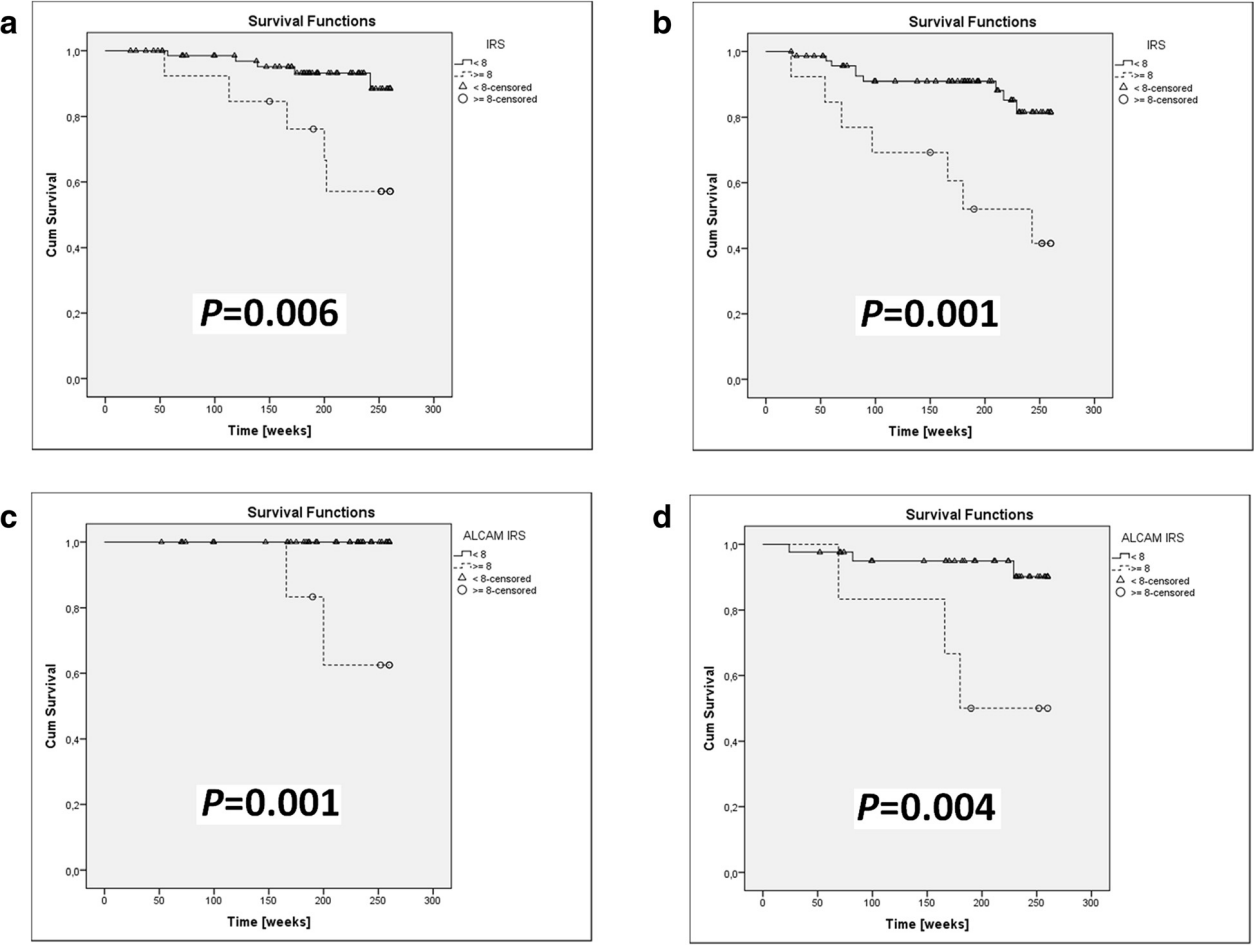
Kaplan-Meier analysis of the impact of ALCAM expression in primary tumors in melanoma patients without regional lymph node metastases (**a, b**) and in patients with negative SLNB status (**c, d**). Survival analysis showed that in patients without locoregional nodal metastases increased ALCAM expression (IRS) is associated with shorter CSOS (**a**) and DFS (**b**); similarly, also in SLNB-negative patients, increased ALCAM expression (IRS) was related to shorter CSOS (**c**) and DFS (**d**)

The expression of ALCAM protein was calculated using a semi-quantitative method. Two immunohistochemical reaction parameters were considered when evaluating the expression of the foregoing proteins: the percentage of cells with a positive cytoplasmic reaction (the percentage of reactive tissue) and the reaction intensity. The final immunohistochemical reaction results are expressed according to the semi-quantitative IRS (ImmunoReactive Score) scale devised by Remmele and Stenger [18]. This scale assigns a score for the percentage of cells demonstrating reaction (0–4 points) and for reaction intensity (0–3 points). The final result is the product of the scores for these two parameters (0–12 points) and is referred to as an IRS factor or score.

### Statistical analysis

Statistical analysis was performed using the Statistica 10.0 and IBM SPSS 21 software packages. Overall survival (OS) was defined as the time between the primary surgical treatment and death, and OS was censored at last follow-up for patients who were still alive. Disease-free survival (DFS) was defined as the time between the primary surgical treatment and the date of relapse. DFS was censored at the last follow-up for patients who survived without disease recurrence or at the date of non-cancer associated death. Cancer-specific overall survival (CSOS) was defined as the time between the primary surgical treatment and cancer-associated death, and was censored at the last follow-up for surviving patients.

A χ2 test, exact Fisher test in the case of 2 × 2 tables, and Spearman rank correlation were used to analyze associations between mitotic rate and the presence of ulceration and clinicopathological parameters. Differences between the means were tested with a nonparametric test (Mann–Whitney *U* test and Kruskal-Wallis test), the log-rank test was used to compare survival in two groups, the overall survival rate was estimated by the Kaplan-Meier method and the influence of explanatory variables on death risk was analyzed by means of the Cox proportional hazard regression. P values <0.05 were considered statistically significant.

## Results

### ALCAM expression in primary tumors and nodal metastases

ALCAM expression, defined as IRS > 0, was identified in 96 primary tumors (92.3 % of cases). In 8 cases (7.7 %) no ALCAM immunoreactivity was detected in cancer cells. High ALCAM expression, defined as IRS ≥ 8 was detected in 18 cases (17.3 %). The mean IRS was 4.39 ± 2.64. Analysis of melanoma cells obtained from nodal metastatic foci showed the presence of ALCAM in 14 out of 16 cases (87.5 %). High ALCAM expression, defined as IRS ≥ 8 was detected in 5 cases (31.3 %). The mean IRS in nodal metastases was 4.69 ± 4.01.

### Correlations between ALCAM expression in primary tumor and lymph node metastases with histopathological features of primary melanoma

Increased ALCAM expression (defined as increased IRS) in melanoma cells in primary tumor is closely correlated with higher Breslow thickness and higher Clark level (*P =* 0.008 and *P* = 0.001, respectively). Similar correlations were observed for a percentage of ALCAM-positive cancer cells, where a higher number of melanoma cells showing ALCAM expression (0-75 % versus 75 %) was closely related with a deeper invasion into the skin layers, both according to Breslow and Clark scales (*P* = 0.003 and *P* = 0.026, respectively) (Table 3).

**Table 3:** Correlations between clinicopathological and histopathological characteristics and ALCAM expression parameters in primary tumors and nodal metastases

	ALCAM expression – primary tumor			ALCAM expression – nodal metastasis		
Clinicopathological parameters	%^a^	Int^b^	IRS^c^	%^a^	Int^b^	IRS^c^
pT^ad^	**0.0001**	0.245	**0.016**	0.257	0.977	0.659
pN^e^	0.211	0.228	0.805	0.439	0.952	0.870
Distant metastases^e^	0.145	0.886	0.152	0.257	0.596	0.749
Recurrence^e^	0.161	0.082	0.705	0.705	0.906	0.669
Age^d^	0.368	0.555	0.303	0.467	0.866	0.793
Gender^e^	0.452	0.596	0.699	0.166	0.724	0.699
Primary tumor location^f^	0.412	0.470	0.586	0.779	0.864	0.992
Histopathological parameters	%	Int	IRS	%	Int	IRS
Breslow thickness^d^	0.003	0.342	0.008	0.340	0.865	0.744
Clark level^d^	0.026	0.912	0.001	0.032	0.598	0.400
Growth phase^e^	0.422	0.714	0.568	100 % vertical growth phase		
Histologic type^f^	0.523	0.082	0.749	0.529	0.906	0.709
Mitotic rate^d^	**0.050**	0.163	0.536	0.834	0.825	0.976
Ulceration^e^	**0.035**	0.795	0.086	0.564	0.840	0.625
Lymphangioinvasion^e^	0.523	0.379	0.480	0.439	0.147	0.274
Microsatellitosis^e^	0.249	0.381	0.616	0.439	0.717	0.444
Tumor-infiltrating lymphocytes (TILs)^f^	**0.010**	0.389	0.451	0.967	0.359	0.549
Tumor regression^e^	0.761	0.364	0.771	100 % without regression		

^a^Percentage of ALCAM-positive melanoma cells (0–4 points)

^b^Intenisty of immunohistochemical reaction (0–3 points)

^c^ImmunoReactive Score (product of the scores for the percentage of positive cells and intensity of reaction (0–12 points)

^d^ *P* value of Spearman rank correlation

^e^ *P* value of Mann-Whitney’s *U* test

^f^ *P* value of Kruskal-Wallis test

Statistically significant results (*P* < 0.05) are in bold font

Statistically significant correlation was also demonstrated between the presence of ulceration and a high percentage of ALCAM-positive cells (*P* = 0.035). A tendency was observed that increased number of cells with ALCAM immunoexpression is correlated with a higher mitotic rate which is an independent bad prognostic factor associated with high proliferative potential of the primary tumor (*P* = 0.05) (Table 3). Interestingly, statistical analysis showed that higher percentage of ALCAM-positive melanoma cells is closely related with decreased intensity of lymphocytic inflammatory infiltration (*P* = 0.01).

Analysis of ALCAM expression in nodal metastatic foci demonstrated that lower percentage of positive cells is closely correlated with deeper infiltration of the melanoma in the primary tumor according to Clark scale (*P =* 0.032) (Table 3).

### Correlations between ALCAM immunoreactivity and clinicopathological parameters

Increased ALCAM expression, defined as higher percentage of ALCAM-positive cells and higher IRS, is strongly correlated with higher extension as per pT parameter identifying the primary tumor (*P* = 0.0001 and *P* = 0.016, respectively). Additionally, enhanced intensity of immunohistochemical reaction related with ALCAM detection shows a trend related with a correlation with cancer recurrence (*P* = 0.082) (Table 3). No other statistically significant correlations were identified between ALCAM expression parameters and other clinical features such as tumor location, age or sex (Table 3).

### Analysis of ALCAM expression effect on 5-year survival in melanoma patients

High ALCAM expression in cancer cells of the primary tumor (IRS ≥8) is closely correlated with unfavorable prognosis in cutaneous melanoma patients as regards cancer specific overall survival and particularly disease free survival (*P* = 0.001 and *P* < 0.001, respectively) (Fig. 2). Additionally, survival analysis showed that a high percentage of ALCAM-positive cells (>75 %) may be considered an unfavorable prognosticator (*P* = 0.007 for CSOS and *P* = 0.025 for DFS) (Fig. 2).

Paradoxically, analysis of 16 cases of lymph node metastases demonstrated a trend correlating shorter CSOS with decreased ALCAM expression (IRS < 8) in metastatic cancer cells. No such correlation was found for DFS (Fig. 2).

In patients without regional lymph nodes metastases (N-) a statistically significant correlation was observed between increased ALCAM expression in the primary tumor and shorter CSOS and DFS (*P =* 0.006 and *P =* 0.001, respectively). A similar correlation was noted for patients with a negative sentinel lymph node biopsy status (SLNB-) (*P =* 0.001 and *P =* 0.004 respectively for CSOS and DFS) (Fig. 3).

### Univariate and multivariable Cox analysis of clinicopathological parameters affecting survival of melanoma patients

Independent bad prognostic factors in cutaneous melanoma patients that were showed to have a statistically significant effect on survival are the recognized clinical parameters: (1) microscopic primary tumor advancement (pT); (2) presence of distant metastasis (pM) and (3) Breslow thickness (Table 4). Interestingly, multivariable Cox analysis revealed the effect of high percentage of ALCAM-positive melanoma cells from the primary tumor as a potential, independent prognostic factor, yet without statistical significance (Table 4).

**Table 4:** Survival prognosticators in cutaneous malignant melanoma patients – univariate and multivariable Cox proportional hazard regression models

Clinicopathological parameters	Univariate models			Multivariable model		
	*P* value	HR	95 % CI	*P* value	HR	95 % CI
Primary tumor (pT)	<0.001	1.896	1.329-2.704	0.001	1.594	1.208-2.103
Distant metastases (pM)	<0.001	7.070	2.648-18.873	0.001	6.406	2.089-19.647
Breslow thickness	0.004	1.161	1.049-1.284	0.033	0.773	0.610-0.980
High percentage of ALCAM(+) cells	0.032	2.317	1.075-4.995	0.079	2.028	0.922-4.463

## Discussion

Our study is the first one to evaluate the effect of increased ALCAM expression on long-term survival in melanoma patients. We demonstrate that high ALCAM expression in primary tumor cancer cells (IRS ≥8) is strongly correlated with unfavorable prognosis as compared with patients with lower ALCAM immunoreactivity in tumor compartment as regards cancer specific overall survival (CSOS) (*P =* 0.001) and disease free survival (DFS) (P < 0.001). Furthermore, the results were similar for a high percentage (>75 %) of ALCAM-positive melanoma cells in primary tumor (*P =* 0.007 and *P =* 0.025 for CSOS and DFS, respectively). An important part of our study was to evaluate the correlation between ALCAM expression in metastatic foci in regional lymph nodes and detailed clinicopathological parameters. It was found that decreased ALCAM expression (IRS <8) in nodal metastases shows a trend related with a correlation with shorter cancer specific overall survival (*P =* 0.083). Additionally lower ALCAM immunoreactivity in nodal metastatic foci was significantly statistically correlated with deeper melanoma invasion in the primary tumor according to Clark scale (*P =* 0.032).

A review of world literature (PubMed; 1970–2014; key words: ALCAM, malignant melanoma) identified only 2 reports that were concerned with analysis of ALCAM expression in tissue material obtained from cutaneous melanoma patients using immunohistochemistry [9, 19].

Van Kempen et al. [9] performed a thorough immunohistochemical analysis of ALCAM expression in 38 benign melanocytic lesions, 55 primary melanomas and 28 metastases (11 originating from the skin, 17 as nodal metastatic foci). It was demonstrated that in the majority of benign lesions (34/38) no ALCAM expression was observed in melanocytes. Interestingly, no ALCAM immunoreactivity was observed in any of the early stage melanomas (Clark I and II). As regards Breslow thickness it was shown that over 70 % of over 1.5 mm thick melanomas had increased ALCAM expression in cancer cells [9]. These results are similar to ours in that increased ALCAM expression in melanoma cells in the primary tumor (defined as higher IRS and a high percentage of positive cells) was strongly correlated with deeper thickness in Breslow scale and higher level in Clark scale. Analysis of ALCAM immunoreactivity in cancer cells from metastases brought interesting results that were similar to ours because van Kempen et al. [9] identified ALCAM expression in 42 % (7/17) of the analyzed nodal metastatic foci while in our study we observed high expression (defined as IRS ≥8) in 5 out of 16 nodal metastatic foci (32 %). Van Kempen et al. [9], due to a different nature of the study (the article was only to present ALCAM expression pattern in benign and malignant melanocytic lesions), did not discuss any correlations with clinicopathological data or their bearing on prognosis.

The study by Klein et al. [19] is the second report on immunohistochemical analysis of ALCAM in melanoma. It evaluates ALCAM reactivity in 71 benign melanocytic lesions, 71 melanomas and 84 metastases. The authors did not specify the type of metastatic foci they examined (nodal or in parenchymal organs). Klein et al. [19] confirmed ALCAM expression in 11/71 of benign lesions, 37/70 of melanomas and 58/84 of metastases. At the same time they observed that in melanomas and metastatic foci ALCAM immunoreactivity was most intense. Klein et al. [19] did not examine correlations with any clinicopathological parameters, nor did they evaluate the effect of ALCAM immunoexpression on long-term survival of cutaneous melanoma patients. The article focuses only on the description of ALCAM expression in a relatively wide spectrum of melanocytic lesions.

Most reports say that homophilic ALCAM-ALCAM interactions are more important in cytophysiology than heterophilic CD6-ALCAM interactions. Interestingly, the study by Swart et al. [20, 21] suggested, based on the current knowledge of intercellular connections, that homophilic ALCAM-ALCAM interactions from a theoretical point of view are generally not necessary for the maintenance of cell adhesion if the proteins of cadherin family and other adhesive glycoproteins work efficiently. What is more, as shown by numerous reports, it is the loss of expression of adhesion molecules that is strongly correlated with cancer progression and formation of nodal and distant metastases. Paradoxically, an opposite situation occurs in the case of ALCAM adhesion protein, where it is the upregulation and increase of homophilic interactions is strongly correlated with melanoma progression (ALCAM paradox). It was showed in in vitro models [12, 14, 15], and our results demonstrating that the upregulation of ALCAM in primary tumor and decreased expression of ALCAM in melanoma cells from nodal metastatic foci as the bad diagnostic indicators is the first report of this kind and is based on the analysis of well-documented clinical material and confirm experimental considerations conducted during studies in cell lines and using laboratory animals [12, 15].

In our opinion the different prognostic significance of ALCAM expression in primary tumor and lymph node metastasis should not be viewed as ALCAM paradox. The involvement of ALCAM in melanoma progression should be seen as composed of two stages [20, 21]. The first stage involves ALCAM overexpression during invasion of the dermis layers (depth of infiltration) by nests of cancer cells (which may be formed as a result of homophilic ALCAM-ALCAM interactions) (local growth stage), while the second stage involves decreased ALCAM expression which facilitates invasion of single cells and formation of metastatic foci in regional lymph nodes, which greatly worsens prognosis. In our study in cancer cells from the primary tumor we observed ALCAM expression in membranous-cytoplasmic and cytoplasmic location, dominantly in cell nests forming specific cytoaggregates, while in cancer cells from nodal metastatic foci the only pattern of intracellular distribution was cytoplasmic expression, which is in line with the considerations of the structural-functional ALCAM analysis (two-stage model of ALCAM expression). It must be stressed that it is a hypothesis that needs to be supplemented and validated empirically with a much larger study group and animal models of metastasis, however, it is a voice in the discussion and confirms the hypotheses concerning the role of ALCAM in melanoma progression.

In spite of large scale multicenter research on cytobiochemistry and oncobiology of ALCAM, numerous questions remain that if answered could help to develop new, promising treatment options and implement a more personalized treatment of melanoma patients.

## Conclusions

Our study is the first one to evaluate the effect of increased ALCAM expression on long-term survival in melanoma patients. High ALCAM expression in melanoma cells of the primary tumor can be used as a marker of negative outcome and may indicate a more invasive phenotype of cancer cells, which would require a more intensive therapeutic strategy. Low expression of ALCAM in regional lymph node metastases is a feature associated with unfavorable prognosis in patients with cutaneous melanoma.

## Abbreviations

ALCAM: Activated leukocyte cell adhesion molecule
CMM: Cutaneous malignant melanoma
CSOS: Cancer-specific overall survival
DFS: Disease-free survival
Fra-2: Fos-related antigen 2
IRS: Immuno reactive score
OS: Overall survival
SNLB: Sentinel lymph node biopsy
TILs: Tumor-infiltrating lymphocytes
ZO-1: Zonula occludens protein-1
